# Staff Perspectives in Mental Health Research Regarding Restrictive Interventions: An Australian Scoping Review and Thematic Analysis

**DOI:** 10.3390/bs14010009

**Published:** 2023-12-22

**Authors:** Jacinta Chavulak, Terry Smyth, Nicholas Sutcliffe, Melissa Petrakis

**Affiliations:** 1 Social Work Department, School of Primary and Allied Health Care, Caulfield Campus, Monash University, Caulfield East 3145, Australia; melissa.petrakis@monash.edu; 2 Mental Health Service, Alfred Health, Melbourne 3004, Australia; t.smyth@alfred.org.au (T.S.); ni.sutcliffe@alfred.org.au (N.S.); 3 Mental Health Service, St Vincent’s Hospital Melbourne, Fitzroy 3065, Australia

**Keywords:** mental health, psychiatry, restrictive interventions, seclusion, restraint, literature, scoping review, thematic analysis

## Abstract

Service users and their families have raised concerns about safety in current acute mental health service delivery. Restrictive interventions are routinely used across mental health settings despite increasing awareness of the negative impacts. Underfunding and risk-averse management practices are implicated as key challenges. Utilizing a scoping review and thematic analysis method, this review explored the existing literature of mental health staff perspectives across various settings (including psychiatric wards and emergency departments), focusing on their experience of restrictive interventions. Four themes were developed: 1. Safety (both staff and patient); 2. Barriers to staff reducing their restrictive interventions; 3. Strength in current practice; 4. Recommendations for change. Key gaps in the literature were the limited perspectives of emergency and crisis clinicians (despite these areas being settings where restrictive interventions are utilized) and limited perspectives from allied health disciplines (despite their employment as clinicians in these settings). It also noted a divide between staff and patient safety, as though these concerns are mutually exclusive rather than cooccurring, which is the experienced reality. Advocacy bodies, governments and the media are calling for a reduction in restrictive interventions in crisis settings. This research synthesis proposes that, to achieve this, clinical staff must be involved in the process and their perspectives actively sought and drawn upon to enable reform.

## 1. Introduction

Internationally, there is a paradigm shift in mental health practice. Over the last twenty years, there has been a groundswell of understanding that restrictive interventions used in acute psychiatric care constitute a breach of human rights [1,2]. The United Nations has called for the protection of the rights of people with a disability, to promote dignity and respect and to reframe the false imprisonment of this cohort [1]. Across Europe, there have been many shifts against restrictive interventions, with many countries banning some forms of these practices and developing a Human Rights Act preventing inhuman treatment [3,4]. In the United Kingdom, there have been various attempts to reduce and ultimately eliminate restrictive interventions with the implementation of best practice models, such as the ‘Safewards’ Model [5]. The Safewards Model has been embraced and is utilized in several countries including Australia with varying results [6]. In the United States, there have been calls to action resulting in legislative change and advocates calling for the Safewards Model and other practices to be implemented to reduce restrictive interventions [7]. Internationally, staff perspectives are being explored, noting the key role staff play in these improvements to service; for example, in Norway, staff perspectives have been utilized to explore ethical challenges in in-patient psychiatric settings [8]. 

Australia is no different, with both local and national legislation reflecting the need to ensure trauma-informed and recovery-orientated practice and the reduction in restrictive interventions [9,10,11].

Restrictive interventions refer to actions guided by health care providers in both hospital and community settings that reduce an individual’s autonomy and choice [9]. They act to control behavior in emergency situations where there is deemed to be an imminent risk of harm to an individual or others and other modes of de-escalation have failed. Interventions can take the form of physical, mechanical and chemical restraint and can occur under the obligations of compulsory treatment within the Victorian Mental Health and Wellbeing Act [10,11] and under the guise of ‘duty of care’ [12]. These interventions are at odds with trauma-informed care [13], the ethos of therapeutic alliance and are known to contribute to physical and emotional trauma in both patients and staff [14].

In Victoria, Australia, government policies aim to reduce restrictive interventions; however, due to safety concerns from clinical staff, there have been challenges in staff retention [9,15]. There has been a call for a further lived and living experience workforce to be embedded within services to support this aim and a call for allyship between clinicians and the lived and living experience workforce [16]. Client-centered and recovery-orientated practice is the accepted ideology when providing care but can be difficult to implement in some settings [17]. 

Given service delivery is conducted by staff, of interest to researchers is staff perspectives of their experience of utilizing and witnessing restrictive interventions in acute adult mental health settings. The researchers collectively have over 70 years of experience working within acute mental health settings and considered it important to engage in research linked to their expertise and practice, with review of the literature a preliminary step to engaging in practice-based research. 

### 1.1. Aims

The aim of this study is to explore the Australian literature regarding multidisciplinary staff perspectives of utilizing and witnessing restrictive interventions in working in adult acute mental health services. By exploring the literature available in this area, policy makers, researchers, staff and patients and their supporters will be able to identify current gaps in practice and the literature to inform these ongoing aforementioned reforms in order to reduce restrictive practices. 

### 1.2. Research Question 

What is the perspective of the current Australian literature regarding multidisciplinary staff perspectives (working in adult acute mental health services) of utilizing and witnessing restrictive interventions?

## 2. Materials and Methods

**Figure 1:**
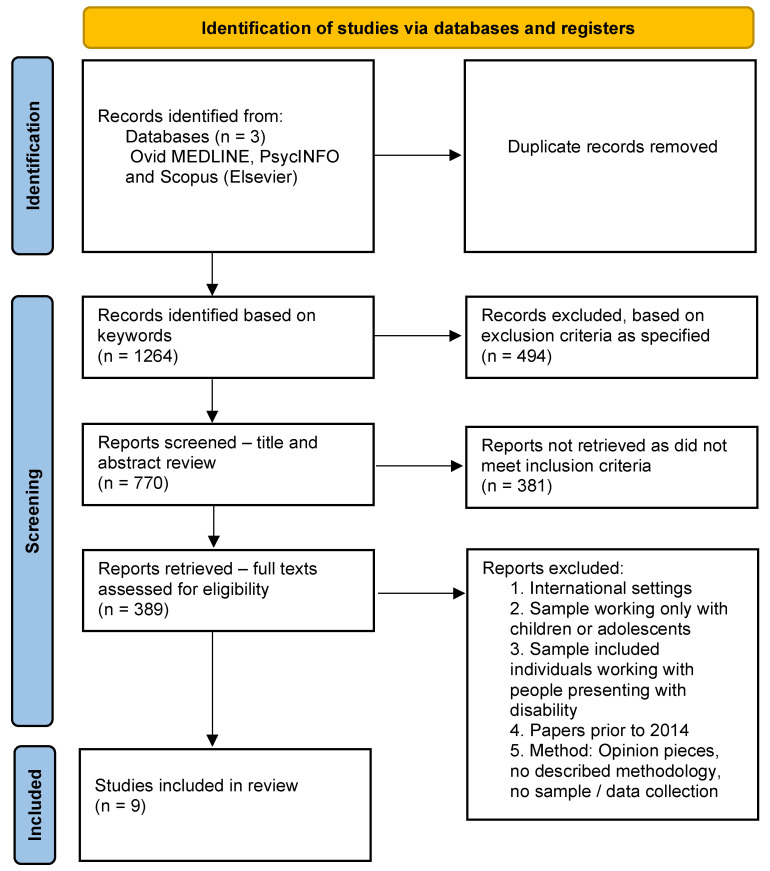
Scoping review PRISMA flowchart [19].

A scoping review of the recent Australian literature was undertaken to explore current knowledge and identify gaps in the literature regarding staff perspectives (see Figure 1). This methodology was deliberately chosen to ensure that a wide range of literature would be initially reviewed to develop an understanding of the current literature available and not be limited by a need to only include randomized control trials and meta-analyses [18]. A scoping review is a ‘preliminary assessment of potential size and scope of available research literature… [which] Aims to identify nature and extent of research evidence (usually including ongoing research)’ [18] (p. 94), which was appropriate given the limited peer-reviewed papers available on this topic. The grey literature was excluded to ensure only peer-reviewed papers with clear methodology were included to ensure papers were of high quality. Australia was chosen as a key area of interest as the clinician-researcher authors work within Australia, and policy is currently shifting to further enhance reductions in restrictive interventions.

Four search terms developed by research team discussion were used: ‘mental health workforce restrictive interventions Australia’/‘psychiatric workforce restrictive interventions Australia’ and ‘mental health restraint workforce perspectives Australia’/‘psychiatric restraint workforce perspectives Australia’. ‘Social work restraint Australia’ was also utilized; however, this registered no appropriate results and is considered a gap in the literature. These were developed based on research team discussion, informed by extensive collective experience in this field. The search was conducted by a university library online search tool, which has access to multiple available databases to ensure a wide variety of literature was obtained. These are listed in the PRISMA table above. 

### 2.1. Inclusion Criteria

1. Papers which were Australian based;
2. Focused on clinical mental health workforce perspectives;
3. Had a methodology which incorporated direct data collection;
4. Related to adults with mental health concerns admitted to emergency departments, psychiatric in-patient units or within community settings.

### 2.2. Exclusion Criteria

1. International setting in which the service delivery occurred;
2. Sample focus was primarily children or adolescents;
3. Sample focus was individuals presenting with disability;
4. Papers prior to 2014, due to a change in the Victorian Mental Health Act at that time [10];
5. Opinion pieces, no described methodology and/or no sample/data collection.

Overall, 1429 participants’ views were included across nine relevant papers [12,13,14,20,21,22,23,24,25]. All papers included perspectives of nurses though not all were practicing and some worked in non-clinical and managerial roles. Four papers included perspectives from other staff; one paper including peer workers [20], one including allied health and medical staff perspectives [21] and two papers with unclear descriptions of the staff designation [22,23]. The vast majority of staff perspectives included were from nurses. Data were collected by interviews, focus groups and surveys. One paper did not explore staff perspectives; it focused on secondary data from code greys and restraints in emergency department (ED) settings relating to 494 patients. It was included as it was the only paper which explored restrictive interventions specifically in emergency departments and would be useful to consider for future research, identifying a gap in the literature. Noting the scoping review method, to ensure an understanding of the situation was developed and presented [18], another key gap in the literature was identified. 

The settings were varied, including perspectives from staff working in psychiatric in-patient settings (including forensic), community psychiatric settings, EDs and some either not specified or post-practice. 

The papers were analyzed utilizing the thematic analysis approach, and the themes developed are outlined below. A thematic analysis allows the results to emerge from the data on a deeper level and allows for research team discussion, insight and interpretation [26] (p. 550). The process utilizes first- and second-level coding [26] and was completed by the first-named and fourth-named (senior editor) authors. 

## 3. Results

### 3.1. Theme 1: Safety

This theme relates to any discussion of safety. 

All papers reported on the theme of safety, both the safety of staff and of service users [12,13,14,20,21,22,23,24,25]. There are two subthemes here: one, a focus on staff safety, and the other, a focus on patient safety. 

#### 3.1.1. Staff Safety

Most papers acknowledged the importance of staff safety. Six papers focused on staff safety as a clear priority, with concerns raised throughout that in the absence of restrictive interventions, staff are left at high risk of assault [13,14,20,23,24,25]. 

One particular area where this was a key concern was forensic mental health settings, where there is an increased level of risk to staff due to a history of violent behaviors [23]. The literature reflected that, at times, staff felt unsafe at work due to the nature of the role [13,14,20,21,22,23,24], with one paper reflecting that fear was a key motivator of decision making:

*“Fear was revealed as a powerful catalyst for decisions around implementing restrictive practices. In this context, fear pertained to the threat of experiencing direct occupational violence. Fear of experiencing violence or of a situation escalating out of control was so strong that participants noted the rights of service users were sometimes seen as secondary to taking an action that was perceived to increase staff safety.”*

[14] (p. 676)

Staff reported routinely facing aggression and assault, with one paper exploring this specifically:

*“Three hundred and twenty-five (83%) participants reported exposure to at least one of the five forms of violence noted in the WHO questionnaire. Three hundred and eleven participants (80%) were victims of verbal abuse, 115 (30%) had been bullied or mobbed, 23 (6%) participants reported sexual harassment and 50 (13%) reported racial harassment. More than a third of participants (n = 132, 34%) indicated having been physically assaulted in the past 12 months and six (5%) of those were with a weapon. The perpetrator was most frequently a patient/client for victims of physical (n = 132, 96%), verbal (n = 285, 69%), racial (n = 47, 73%) and sexual (n = 24, 75%) violence/harassment.”*

[23] (p. 447)

Papers noted the psychological impact on the staff themselves [12,20] and noted that restrictive interventions and the circumstances leading to these were a profound source of occupational distress [14]. There were reports of an increased level of acuity on the wards, which impacts the health and safety for staff, as well as the high number of turnover of staff, impacting on their ability to engage and de-escalate appropriately, heightening their risk of assault [25]. One paper reflected on the importance of trauma-informed care and person-centered treatment in these settings but that this was sometimes at odds with the nursing role of risk mitigation and the way services have been developed [25]. It explored the tightrope walk of ward nurse work:

*“However, there was conflict within the nursing role with nurses expected to both provide person-centred care and be responsible for the safety of all consumers, staff, and visitors: … .you’ve got a duty of care to 30 people or human rights for one. (FG5) In attempting to balance these roles, nurses erred on the side of caution; ‘it’s always about safety’ (FG2).”*

[25] (p. 1514)

#### 3.1.2. Patient Safety 

All papers noted that the safety of patients was of upmost priority for staff and acknowledged the trauma that patients experience. Four papers take a stronger focus on this as a priority [12,20,21,22]. Two of these papers included views of staff participants with a lived experience [20,22]. 

One paper called for the elimination of seclusion and restraint and noted a shift in staff perspective from the previous literature:

*“Our findings may be indicative of an increased willingness among practitioners to change their use of restrictive practices; an enhanced appreciation of how harmful restrictive practices have been for some consumers may enable further support for those seeking change.”*

[22] (p. 541)

Another noted a current gap in the protection of rights of patients when they are held under duty of care and noted that processes were not as rigorous [12]. 

One paper explored a process introduced to protect patients and improve safety for them during restraint; however, it noted that it may in fact increase their risk of injury. For example, staff may be rushed if there is a time limit introduced, associated with the limitations of measures trying to reduce restraints at times as they have adverse outcomes for the safety of patients:

*“The placement of a time restriction (‘2-min rule’) on prone restraint was particularly concerning for staff who questioned the research around this specific time limit. They felt that a time restriction could lead to the restraint process being rushed with an increased risk of errors. It was noted that the duration of restraint was dependent on the level of violence and resistance presented by the individual patient and in many situations, it may not be practical to cease the restraint within a specified time limit.”*

[20] (p. 894)

The trauma and re-traumatization of patients was well documented in current studies and was noted in the past literature [20,22]. Although trauma-informed care is a well-established theoretical concept underpinning current policy, it is not always translated to practice [20]. 

A paper reviewed their own new program, which aimed to have a specially trained de-escalation team present to support patients during their time of escalation, though staff had mixed responses to this [21]. 

Safety, both staff and patient, was a key area of interest across all papers from staff, though papers had various perspectives on what constituted safety (for some, restrictive interventions provided safety, for others, the use of restrictive interventions were a risk to safety). 

### 3.2. Theme 2: Barriers to Staff Reducing Their Restrictive Interventions

This theme related to any barriers noted by staff to reduce their restrictive interventions. 

All papers included a variety of barriers to reducing restrictive interventions that were raised by staff and supported by the previous literature in this field [12,13,14,20,21,22,23,24,25].

The environment, setting and circumstances in which someone is hospitalized impacts on their likelihood to engage in behaviors of concern [21,24,25]. Building design was raised in two papers [21,25], noting the current in-patient unit settings are a barrier for safe de-escalation. The environment was noted as a key source of risk:

*“Many of the participants lamented the building, which had no access to outside space for the patients upstairs, no appropriate rooms in which to separate patients from each other except the bedrooms, and much of the space was in poor condition. Having no choice but to remain in a room with other patients with significant problems was disturbing and un-therapeutic. There were few options for limiting sensory load on patients or removing them from a confronting situation.”*

[21] (p. 894)

An increase in drug-affected service users correlated with an increase in behaviors of concern [12,21] in both emergency department and IPU settings. Fear, as a theme, arose in studies [14,25]; research noted that often restrictive interventions were in response to fear [14], and there was a fear of practices being removed without anything to replace them [25].

Staff noted feeling blamed for restrictive interventions, though noted attempting to follow current policies and guidelines in their service settings:

*“This interconnection of fear and blame ultimately undermines the imperative to eliminate seclusion and restraint in mental health care.”*

[25] (p. 1518)

Conditions for staff, including a high staff turnover, increased burnout and staff shortages, all correlate to an increase in restrictive interventions [25], as well as noted impacts on the ability to develop appropriate rapport [20,25]. A lack of resources for the sector was noted as a factor in restrictive interventions [13]. 

Papers relating to this theme noted key barriers to reducing their restrictive interventions, and solutions to this were explored in theme 4 below. 

### 3.3. Theme 3: Strengths of Current Practice

This theme related to the noted strengths of staff in their current practice, as identified by staff. 

Papers emphasized the strengths of the highly skilled clinicians in this area and noted the importance of this expertise in a high-pressure environment [13,21,24]. These skills/strengths were integral to reducing restrictive interventions in their workspace, and these three papers noted the importance of these skills in the delivery of service. 

A key strength highlighted was staff experience and confidence in de-escalation and responding to aggression:

*“Respondents indicated that there was potential for threatening situations to occur on their unit… Despite this, respondents were confident in their abilities to handle consumer aggression or hostility.”*

[13] (p. 217)

Lower rates of restrictive interventions were linked with good therapeutic rapport [13,22] and staffs’ capacity to recognize escalating behaviors and engage in de-escalation strategies. [21]. One paper evaluated a new process in a psychiatric IPU to help manage aggression and it noted recognition of these behaviors as a key strength:

*“The signs of impending problematic behaviour were not the same for each patient. The ability to see and interpret changes in patient behaviour and intervene in the early stages were core skills in treating and managing aggression.”*

[21] (p. 891)

Experience in the location and with the specific patient group was also noted to be a key area of strength, for example, those working in forensic mental health explored their knowledge of the patient as a key area of strength:

*“A specialist skill identified was possessing knowledge and an understanding of how offending behaviours may be manifested in people during an acute phase of their mental illness. Participants described the need to separate the criminal issues from the person’s mental illness.”*

[24] (p. 892)

Specific personable skills also were noted to have a positive influence on their practice and were identified as key skills:

*“Displaying confidence, having a non-judgemental attitude, and demonstrating flexible boundaries to accommodate the wide range of patient behaviours were identified as an important clinical skill in preventing and managing aggression whilst maintaining the therapeutic environment.”*

[24] (p. 892)

Overall, papers which included this theme noted the high level of specific skills needed to practice in their area. 

### 3.4. Theme 4: Reccomdations for Change/Improvement

This theme related to any recommendations from the papers to change, derived from the staff perspectives outlined—for example, changes to policy, changes to practice or implementation of additional programs. These are recommendations from their specific settings.

As sample working settings varied, there were also varied recommendations for improvement. All agreed that reducing restrictive interventions was a key goal, although had varied proposals about how this would be achieved and varied responses regarding if it could be achieved. One paper recommended improving capacity for training to ensure any new workforce were adequately trained prior to commencement of work [24]. Studies noted the importance of de-escalation strategies [13,20] and one study in particular recommended that specific de-escalation teams be introduced across all mental health IPUs [21]. Consensus was difficult with the research reviewed highlighting the difficulty of eliminating restraint due to the complex nature of behaviors of concern and the limited alternatives offered currently [13,20,21,23], with one calling specifically for the elimination of restraint [22]. One paper highlighted the delicate nature of this balance, suggesting that without specific alternatives offered by services, elimination will remain difficult:

*“In spite of calls for the reduction and elimination of seclusion, physical restraint, and mechanical restraint reflected at the policy or research level, these practices are still used in Australia and nurses hold mixed beliefs regarding their elimination. Nurses do not necessarily see the practices as favourable, but necessary for maintaining a safe work environment. … At a wider level, the present findings highlight the importance to seclusion and restraint reduction and elimination efforts of strong clinical leadership, sufficient staff numbers and resources, consideration of the appropriateness of the physical unit environment, and appropriate resources for the use of alternative methods to seclusion and restraint that maintain staff and consumer safety. In addition, a focus on trauma-informed care, empathic relating to consumers, training/education of staff, and team collaboration and cohesion are essential to reduction efforts.”*

[13] (p. 222)

One paper called for a change in the system of governance from duty of care to more stringent acts, similar to MHA, for all patients in acute settings [12]:

*“A framework for the governance of restrictive interventions in acute settings must be developed. It should apply consistently to all patients, not just a proportion managed under Mental Health legislation. The framework should consider consumer, organisational and staff perspectives. It would include continuous quality improvement and focus on minimizing the rates of restrictive interventions while maintaining a safe environment for patients, staff and visitors.”*

[12] (p. 399)

Two papers specifically noted the importance of considering staff safety during any period of reform [14,24] and called for appropriate staffing levels and debriefing, as well as the need to support staff due to the trauma experienced by the implementation of such practices and the circumstances precipitating these interventions [14]. 

No paper stated that restrictive interventions were favorable or positive, but rather they were something that needs to have a narrow criterion of use (if used at all), and their place in future systems needs to be rigorously scrutinized. No paper stated that the current practice was perfect and all had some recommendations for future practice. 

## 4. Discussion

In the literature, themes of safety for patients and staff were presented as mutually exclusive, thus re-enforcing the idea of two distinct groups within these settings. To clarify, staff often feel that the behaviors of patients put their safety at risk; however, they also acknowledge that restrictive interventions (a way of managing these behaviors as per hospital policy) infringe on the safety of patients. This can be divisive and unhelpful when developing reforms. The data developed saw both staff and patient safety as important components of an effective health service. On a somewhat contradictory note, clinicians often walk on a tightrope; staff are represented as advocates for the reduction in restrictive interventions and are also a group that would face a greater risk to their safety without its continued use. 

None of the articles located included clear social work perspectives, with only one paper [21] referring to allied health despite social workers being employed as clinicians within crisis teams across Victoria. This has identified a gap in the literature for future research to consider. For the study location chosen, allied health makes up approximately one third of the team, with the majority of these being social workers. 

Most of the articles focused on psychiatric in-patient settings, with only one paper [12] focusing solely on the emergency department (ED) as their setting (another paper had participants [13] who worked in either IPU or ED settings but they were not distinguishable in their results). This paper did not specifically refer to psychiatric patients; however, it was included as it referred to behaviors of concern and discusses a setting in which mental health crisis workers often are engaged in restrictive interventions (the chosen sample for the current study). Emergency departments see a significant proportion of those seeking psychiatric care in crisis situations, including situations which currently require the use of restrictive interventions both under compulsory treatment (for example, in Victoria under the Mental Health and Wellbeing Act [11]) and under “duty of care” frameworks [12]. This review highlights the disproportionate lack of data available on how safety risks are managed across disciplines in this setting. 

The Royal Commission into Victoria’s Mental Health System (RC) [27] has placed significant emphasis on shifting psychiatric care from EDs and notes the issues with these settings becoming an entry point for psychiatric care, with psychiatric workers in these settings facing burnout and fatigue. The RC calls for a reduction in restrictive interventions in this setting; however, there appears to be limited research in this area specific to the psychiatric workforce within ED settings and community crisis teams. Another area needing further research is studies focusing on the perspectives of the vast array of staff disciplines who also work in this field, such as social work, as well as the psychiatric nursing staff views reflected in this review. 

### 4.1. Implications for Practice/Research/Policy

Considering the recent findings of the Royal Commission into Victoria’s Mental Health System (RC) [27], this review is an important step in including staff perspectives in the development of a new mental health system. The media, recent Victorian Mental Illness Awareness Council (VMIAC) research [28], service user bodies and the RC all emphasize the need for services to reduce (and some calls, to eliminate) restrictive interventions. The National Mental Health Workforce Strategy [29] and Victoria’s Mental Health and Wellbeing Workforce Strategy 2021–2024 [15] explore a number of barriers to ensuring these important interventions are carried out, including staff retention. The current literature reviewed acknowledges the limitations of staff, from funding, risk aversive policy and appropriate environment design. Utilizing these strategies as a guide, this review aims to pave the way for clinicians to be included in future research in this area so they can contribute to improving health systems in crisis and emergency settings.

### 4.2. Limitations

The authors note the small sample obtained, demonstrating the limited papers in Australia focusing on staff perspectives in this area. The methodology was specifically chosen to include a wide range of literature, and an even smaller sample would have been obtained should the methodology be amended to a more rigid option such as a meta-analysis [18]. 

## 5. Conclusions

The literature reviewed presents varying perspectives; some research emphasizes staff safety, presenting a sense that this is at odds with restrictive interventions, while other research emphasizes the adoption of a human rights approach for working with patients. Throughout the available literature, there appears to be a consensus that the application of restrictive interventions does have adverse impacts on staff and patients alike. The competing paradigms and emphasis between risk aversion-based practices and recovery-orientated practice evident throughout the literature had practice implications, with staff often expressing stress at the incongruities in the expectation to implement both at once. 

The literature suggests that health and legal systems, environmental factors and resourcing issues frequently limit a clinician’s ability to take positive risks and support each patient’s rights. Overall, there exists a paucity of literature with regard to clinician’s perspectives in the use of restrictive interventions, with prominent gaps in perspectives from both the emergency department setting and the allied health workforce.

## 6. Future Directions 

Current research and policy have advocated for reducing restrictive interventions, but there has been limited scope for those who work in these fields to actively participate in improving the mental health system. This scoping review was conducted to clarify current practice prior to future practice-based research to explore barriers and enablers to congruent clinical leadership in practice, to inform policy directions and to strengthen the responsiveness to enacting espoused intentions in reform. 

## Data Availability

Data is unavailable due to privacy; the correspondence author can be approached with specific requests.
